# Supplementary material for: Investigating microbial population structure and function in the chicken caeca and large intestine over time using metagenomics
Source: BMC Res Notes. 2025 Aug 15;18:355. doi: 10.1186/s13104-025-07441-7 (PMC12357378; doi:10.1186/s13104-025-07441-7)
Supplement: Supplementary file 2 — Recovered nutrient cycles including Carbon, Nitrogen, and Sulphur cycles for all the metagenomic assembled genomes given as PDF images, and identifiable through bin numbers. [file 13104_2025_7441_MOESM2_ESM.zip › Supplementary Data2/bin.12.draw_carbon_cycle_single.pdf]

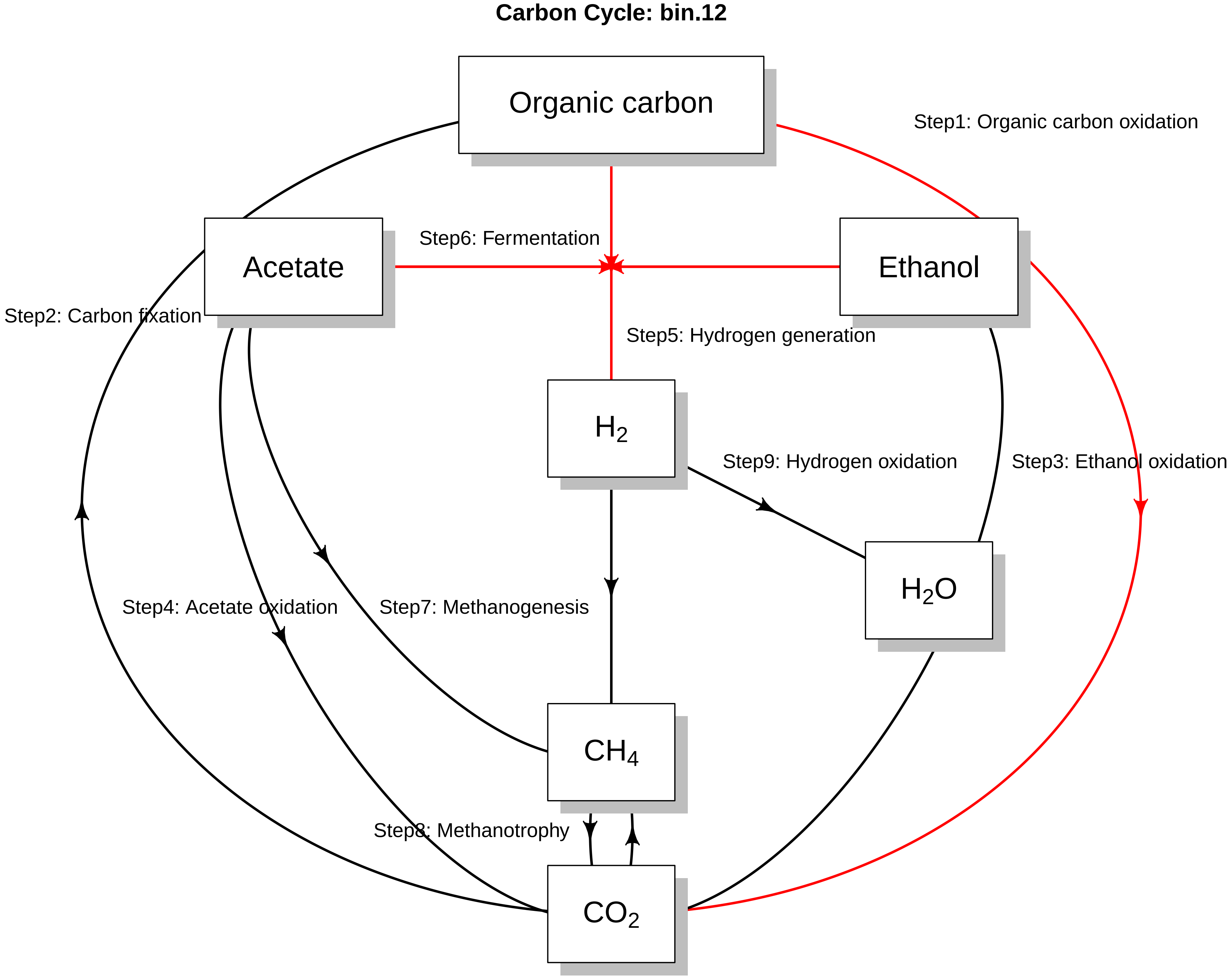

Carbon Cycle: bin.12
Organic carbon
Step1: Organic carbon oxidation
Acetate
Step6: Fermentation
Ethanol
Step2: Carbon fixation
Step5: Hydrogen generation
H_2
Step9: Hydrogen oxidation
Step3: Ethanol oxidation
H_2O
Step4: Acetate oxidation
Step7: Methanogenesis
CH_4
Step8: Methanotrophy
CO_2
